# The role of melatonin in the treatment of type 2 diabetes mellitus and Alzheimer's disease

**DOI:** 10.7150/ijbs.66871

**Published:** 2022-01-01

**Authors:** Shengnan Shen, Qiwen Liao, Yin Kwan Wong, Xiao Chen, Chuanbin Yang, Chengchao Xu, Jichao Sun, Jigang Wang

**Affiliations:** 1 Artemisinin Research Center, and Institute of Chinese Materia Medica, China Academy of Chinese Medical Sciences, Beijing, China.; 2 State Key Laboratory of Quality Research in Chinese Medicine, Institute of Chinese Medcal Sciences, University of Macau, Taipa, Macau, China.; 3 School of Life and Health Sciences, The Chinese University of Hong Kong, Shenzhen, Guangdong, China.; 4 Department of Pharmacology, Yong Loo Lin School of Medicine, National University of Singapore, Singapore 117600, Singapore.; 5 School of Biopharmacy, China Pharmaceutical University, Nanjing 211198, China.; 6 Department of Geriatrics, Shenzhen People's Hospital (The Second Clinical Medical College, Jinan University; The First Affiliated Hospital, Southern University of Science and Technology), Shenzhen, China.; 7 Shenzhen Mental Health Centre, Shenzhen Kangning Hospital, Shenzhen, China.; 8 Guangdong Provincial Key Laboratory of New Drug Screening, School of Pharmaceutical Sciences, Southern Medical University, Guangzhou, China.; 9 Key Laboratory of Prevention and Treatment of Cardiovascular and Cerebrovascular Diseases, Ministry of Education, Gannan Medical University, Ganzhou, China.; 10 Central People's Hospital of Zhanjiang, Zhanjiang, Guangdong, China.

**Keywords:** Melatonin, Type 2 diabetes, Alzheimer's disease, Mitochondria, Anti-inflammatory, Insulin resistance

## Abstract

In type 2 diabetes mellitus (T2DM) and its related disorders like obesity, the abnormal protein processing, oxidative stress and proinflammatory cytokines will drive the activation of inflammatory pathways, leading to low-grade chronic inflammation and insulin resistance (IR) in the periphery and impaired neuronal insulin signaling in the brain. Studies have shown that such inflammation and impaired insulin signaling contribute to the development of Alzheimer's disease (AD). Therefore, new therapeutic strategies are needed for the treatment of T2DM and T2DM-linked AD. Melatonin is primarily known for its circadian role which conveys message of darkness and induces night-state physiological functions. Besides rhythm-related effects, melatonin has anti-inflammatory and antioxidant properties. Melatonin levels are downregulated in metabolic disorders with IR, and activation of melatonin signaling delays disease progression. The aim of this Review is to highlight the therapeutic potentials of melatonin in preventing the acceleration of AD in T2DM individuals through its therapeutic mechanisms, including antioxidative effects, anti-inflammatory effects, restoring mitochondrial function and insulin sensitivity.

## Introduction

**Figure 1:**
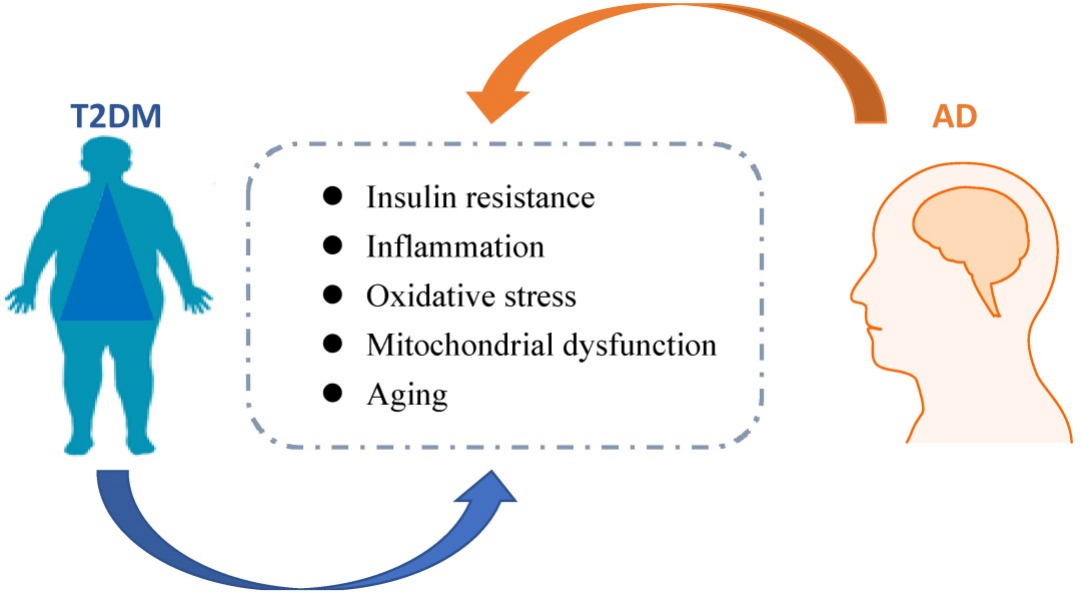
** The link between AD and T2DM.** Pathological mechanisms associated with T2DM might accelerate AD progression. Insulin resistance, inflammation, oxidative stress, michondrial dysfunction, and aging are related to diabetes, which possibly contributed to AD development.

Metabolic disorders, including type 2 diabetes mellitus (T2DM) and obesity-related insulin resistance (IR) accelerate not only cerebrovascular disease and stroke, but also neurodegenerative diseases, especially the development of Alzheimer's disease (AD) 1, 2. It is generally accepted that brain IR leads to failure of response to insulin, eventually causing impairments in metabolic and immune functions. Globally, the epidemics of T2DM and AD are increasing, which bring huge costs in economic burden and human life suffering. Hence, it is necessary to develop preventive or disease-modifying agents based on pathological studies of these diseases. The link between T2DM and AD was shown in Figure 1.

Exogenous melatonin has been investigated as a therapeutic agent for many diseases. Notably, the metabolic effects of melatonin in T2DM and obesity have been of interest 3, 4. Also, decreased melatonin production and secretion were shown to be related to neurological diseases like schizophrenia, stroke, and AD 5, 6. Besides the various physiological roles of melatonin in endocrine and neurological disorders, numerous actions of melatonin have been proven safe in animal models and human beings at a wide-range dosage window. In this review, the therapeutic efficacy of melatonin in reversing IR on T2DM and AD will be discussed. Moreover, whether melatonin will be beneficial in halting AD progression in T2DM individuals will be summarized. We hope this review will highlight the therapeutic potentials of melatonin for the treatment of T2DM and AD.

T2DM is one of the risk factors for AD onset. The underlying mechanism is suggested to be the dysfunction of insulin signaling 7. Insulin resistance was also observed in AD patients that did not have T2DM 8. The insulin receptor is responsible in enhancing glucose uptake, mitochondrial function and replacement, anti-apoptosis, and autophagy via MAPK, AKT signaling pathways, and Nrf2 activation against oxidative stress 9-11. Therefore, Insulin is not only a hormone for glucose homeostasis, but also a key regulator in neuronal generation, repair, and functions 12.

## Association between pathogenesis of T2DM and AD

High levels of blood glucose elevate the risk of dementia in both diabetic and nondiabetic individuals by rates of 40% and 10%, respectively 13. Obesity, a T2DM-related disease, has also been shown to increase the risk of AD and dementia in the elderly population 14 In the following section, the pathological links between T2DM and AD, especially the mechanisms of the development of T2DM to AD including inflammation and defective insulin signaling, will be summarized.

### Inflammation

**Figure 2:**
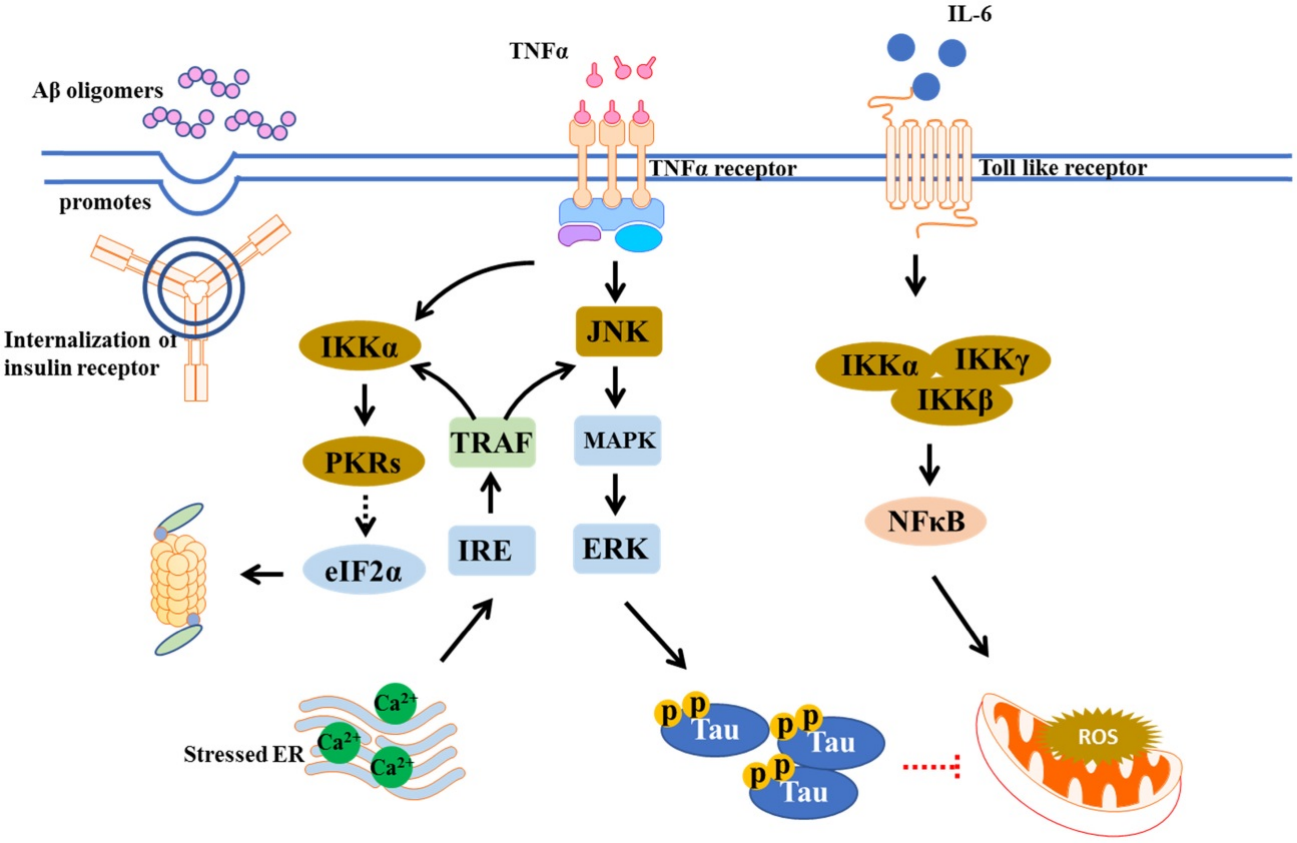
** Overlap inflammation signaling in AD and T2DM.** Microglial activation by Aβ oligomers stimulates production/release of TNF-α. TNF-α receptor activation promotes stress kinases including JNK, IKK, and PKRs, which in turn blocks the insulin actions.

Chronic inflammation is thought to participate in the pathogenesis of T2DM. In metabolic disorder like T2DM, the adipose-resident macrophages are polarized towards a pro-inflammatory (M1-polarized) phenotype, increasing the expression of inflammatory mediators, including interleukin-6 (IL-6), tumor necrosis factor-α (TNF-α), and IL-1β. These proinflammatory cytokines can cross the blood brain barrier, causing brain insulin resistance 15. Notably, inflammation also underlines hypothalamic dysfunction in obesity 16. It was found that insulin receptor substrate 1 (IRS-1) inhibition by amyloid β (Aβ) oligomers, a pathological hallmark of AD, via TNF-α/JNK activation showed impaired brain insulin signaling in AD and promoted proinflammatory signaling 17. The IkB kinase (IKK), a stress kinase, was found to be activated by TNF-α in peripheral metabolic tissues and AD brains 18. Recently, a multiplexed immunoassay revealed neuroinflammatory changes along with diabetic symptoms using different models, including APPswe/PS1dE9 (APP/PS1) mice with high-fat diet (HFD), APP/PS1 with db/db mice, and APP/PS1 with STZ, which found that the levels of both chemokines like MIP-1α, MIP-1β, and MCP-1, and proinflammatory cytokines like Il-1α, Il-3, and IFN-γ were upregulated in these AD pathology-associated T2DM models 19. The broad range of cytokines promoted neuronal injury, BBB breakdown, and brain insulin resistance. The peripheral mediators including cytokines and adipokines may link the peripheral and central inflammatory, as shown in Figure 2, so the approaches which combat these dysregulated signaling events may have the potential to treat T2DM and AD.

Neuroinflammation can be triggered by Aβ deposition and tau hyperphosphorylation 20. It is involved in microglia activation that primarily targets Aβ phagocytosis. However, sustained microglial activation leads to accumulation of inflammatory mediators and danger-associated molecular patterns (DAMPs), limiting Aβ clearance, resulting in more plaque accumulation and neuronal dysfunction 21, 22. Inflammation is suggested to be linked to insulin resistance. Insulin resistance can increase levels of advanced glycation end products (AGEs), which cause upregulation of GSK-3β and activation of NF-κB pathway, thus induces ROS and pro-inflammatory cytokine production 23. These pro-inflammatory cytokines were observed to inhibit phagocytosis thus enhance Aβ accumulation, while NF-κB signaling pathway activates AGEs binding to in turn increase Aβ expression 24, 25.

### Defective insulin signaling

**Figure 3:**
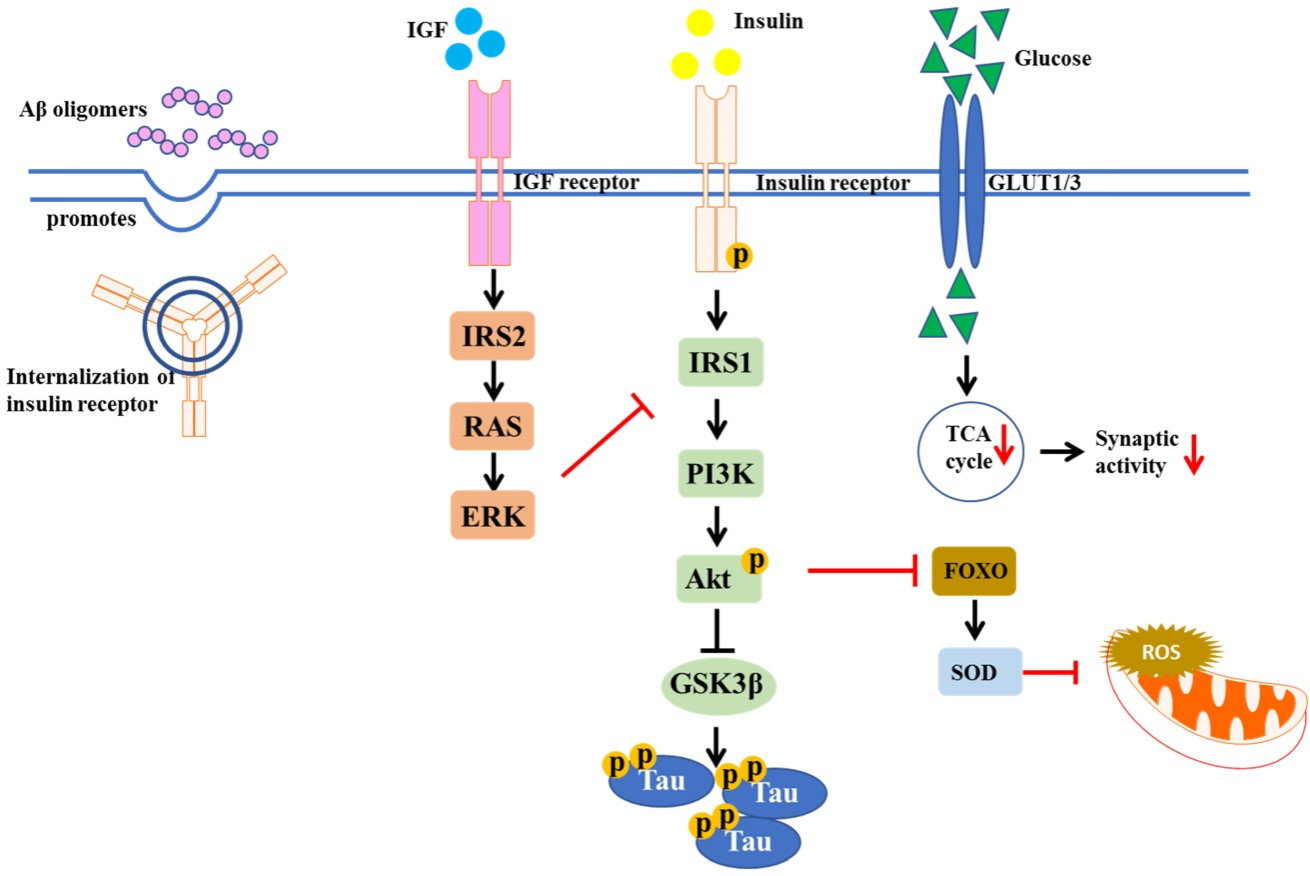
** Insulin signaling pathway implicated in T2DM and AD.** The diagram shows the defective insulin signaling. IR may result from impairment of insulin receptor function, tyrosine dephosphorylation of insulin receptor and IRS, as well as the disturbance of glucose transportation, which in turn decreases synaptic activity.

In both human and rodents, high dietary fat intake could increase oxidative stress and ROS production in skeletal muscles, leading to the development of peripheral IR in T2DM 26. IR is a hallmark of obesity and T2DM, which is also found in the brains of AD patients 1. Indeed, many studies suggest that the incidence of AD is higher in obesity and T2DM patients. In obesity and diabetes, the signaling pathway IR/insulin-like growth factor (IGF) was altered 27. Insulin-degrading enzyme (IDE) is important for insulin and Aβ clearance. IR can lead to hyperinsulinemia, which saturates IDE for insulin and Aβ degradation. Thus, dysfunction in Aβ degradation caused by IR increases risk of AD onset 28. In AD brains, over-activation of N-methyl-D-aspartate (NMDA) receptors by Aβ oligomers is a key factor resulting in excessive ROS production, followed by excessive Ca^2+^-induced mitochondrial dysfunction. Brain insulin signaling acts to block Aβ oligomers-induced neuronal oxidative stress, via activation of AKT and prevention of aberrant NMDA receptor signaling 29, 30. Moreover, Aβ oligomers desensitize the insulin receptors from plasma membrane in cultured hippocampal neurons, reducing tyrosine kinase activity of the insulin receptor protein, which is important for tyrosine phosphorylation and subsequent activation of insulin receptor substrates (IRS), like mTORC1, PI3K, and Akt 31. The IR in the brain is shown in Figure 3. Therefore, the agents which can stimulate brain insulin signaling may facilitate neuroprotection in AD and preserve normal brain functions.

Insulin signaling is essential in proper brain function like memory formation. Impaired brain insulin signaling can cause cognitive decline in human and animal models. Post-mortem AD displayed hyperphosphorylated Tau-containing neurons and insulin accumulated tauopathies 32. Conversely, insulin resistance can in turn induce Tau hyperphosphorylation. Compared to normal individuals, higher levels of phosphorylated Tau in cerebrospinal fluid (CSF) were observed in cognitive dysfunction subjects due to systemic insulin resistance 33. A mechanism underlying this phenomenon involves a Tau kinase, the glycogen synthase kinases 3β (GSK3β), regulated by insulin through AKT pathway 34. Insulin resistance due to chronic exposure of high insulin levels of neurons, or eventual decrease in insulin levels in brain, reduces AKT phosphorylation, leading to an activation of GSK3β, inhibition of Tau phosphatases, ultimately Tau phosphorylation 35, 36.

## Physiological roles of melatonin

Melatonin is essential in the management of circadian rhythms of healthy metabolism. There are two specific receptors of melatonin, MT1 and MT2, encoded by MTRN1A and MTRN1B, respectively 37. When melatonin binds to MT1 and MT2, the subunits α and β/γ dissociate to trigger downstream signaling pathways including adenylyl cyclase (AC), phospholipase C (PLC), and phospholipase A2 (PLA2) 38. It has been shown that disturbance of melatonin signaling is implicated in development of T2DM raised by IR 39-42. Impairment of sleep and circadian systems are involved in T2DM and obesity etiology, suggesting the prevalence of metabolic disorders in the individuals with irregular lifestyle like light at night, night-shift working, unusual meal timing, are increasing 43, 44. Thus, a combination of the chronobiotic and cytoprotective effects of melatonin may be an innovative strategy in T2DM treatment. The beneficial effects of melatonin on different models of T2DM are shown in Table 1.

### Satiety and appetite regulation

Melatonin is important for the secretion of metabolic hormones leptin and ghrelin to regulate satiery and appetite. Leptin is a regulator for the anorexigenic response. In hypothalamic neurons, it manages energy homeostasis via activation of leptin receptors (LepR), followed by activation of Janus kinase2 (JAK2) and signal transducer and activator of transcription 3 (STAT3) pathways 63, 64. Increasing levels of leptin can down-regulate the adipose mass while leptin resistance may occur in obesity individuals. Recently, it was shown that MT1 signaling could modulate leptin signaling. Rats with melatonin deficiency were observed to have leptin resistance and increased body weight, and these defects were reversed with melatonin administration 65, 66. MT1 knock-out mice showed more daily food intake that led to increased body weight compared with the wild type mice, indicating that MT1 is critical in feeding behavior^53^.

Ghrelin plays an essential role in orexigenic behavior. Plasma levels of ghrelin elevate before each daytime meal, decrease after mealtime, and increase progressively during fasting overnight, demonstrating that ghrelin release triggers appetite initiation. Growth hormone secretagogue receptor (GHSR) activated by ghrelin up-regulates the intracellular levels of PIP3 and Ca^2+^ via triggering PLC and PKC 67. Increasing Ca^2+^ influx activates hypothalamic calcium/calmodulin-dependent protein kinase kinase 2 (CaMKK2), followed by AMPK activation 68, 69. Ghrelin exhibits rhythmic secretion under feeding or fasting conditions. An immunohistochemical study showed that rats which had undergone pinealectomy almost completely abolished ghrelin secretion in the arcuate nucleus (ARC) region 70. However, ghrelin levels in the plasma of exogenous melatonin treatment or removal of pineal gland were not significantly different from the control group. Thus, it is considered that the interaction between ghrelin and melatonin may be indirect. As mentioned above, serotonin acts as a melatonin precursor and mediates the regulation of appetite, thermogenesis, and higher level of mental functions like memory and learning. It is possible that ghrelin attenuates melatonin release by disrupting serotonin biosynthesis and secretion from pineal gland.

### Circadian clock and food intake

Animals can sense the time of food availability. The ghrelin, glucocorticoids, and glucagon secreted before mealtime are classified as pre-feeding timers. Meanwhile, hormones like insulin and leptin which are secreted after food intake are called post-feeding timers 71. Avoiding excessive energy intake during rest phase is critical for healthy metabolism 69. Although melatonin is not strictly recognized as a metabolic hormone, melatonin plays important roles in glucose homeostasis. Melatonin can indirectly modulate feeding behavior on the circadian clock. In mice and rats, melatonin administration decreases adipose mass and body weight 72. In diet-induced obesity zebrafish, melatonin stimulated the anorexigenic and inhibited the orexigenic signaling 73. Thus, the lesion of circadian oscillations may disturb the control of energy balance, thus causinging metabolic diseases like obesity and T2DM.

### Mitochondria biogenesis and bioactivity

As previously mentioned, melatonin is an ancient antioxidant. As for the subcellular distribution of melatonin, the rank of its concentrations from high to low is mitochondria, cell membranes, nuclei, and cytosol in brain 74. It is reasonable that antioxidants such as melatonin are efficient in decreasing total oxidative burden, as ROS is highly produced in mitochondria. In addition, it is proposed that melatonin is highly effective as a mitochondria-target antioxidant 75, 76. The potency of classic antioxidants being limited even in high doses may be due to their difficulty in accessing mitochondria. Therefore, melatonin can be a good candidate to increase the therapeutic effectiveness via anti-oxidative activity.

Mitochondrial dysfunction may accelerate the AD onset that accompanies aging 77. A key direct association between aging and mitochondrial function was observed in many models. In rats, aging leads to brain mitochondrial dysfunction, comprising of changes in expression levels of mitochondrial genes and decreased activities of respiratory chain related enzymes 78. Also, age-related mitochondrial impairment was observed in amyloid-based transgenic mouse models, which act through inhibition of oxidative phosphorylation 79, 80. Melatonin increased mitochondrial bioactivity, which subsequently attenuated Aβ accumulation and synaptic dysfunction and exhibited neuroprotective effects in AD mice 81, 82. Also, melatonin administration was effective in AD models through triggering free radical scavenging cascades 83, 84. Melatonin treatment on APP/PS1 mice was elucidated to restore the membrane potential, mitochondrial respiratory rates, and ATP levels in cortex, hippocampus, and striatum 85. These evidences suggest melatonin or activating melatonin receptor signaling can be a potential strategy in delaying AD progression.

The circadian clock is important in metabolism according to both human epidemiological and interventional studies. Disruptions of circadian genes lead to striking metabolic disturbances 86. It is only recently understood that circadian disruption might contribute to diabetes and β-cell dysfunction 87. In T2DM rats, melatonin supplementation combined with exercise showed increased expression of mitochondrial biogenesis and function-related genes, including mtTFA, PGC1-α, NRF-1, and NRF-2. Moreover, melatonin intake combined with exercise is effective in scavenging toxic free radicals, suggesting melatonin administration showed anti-diabetic effects via anti-oxidative pathways 53. Recently, melatonin was shown to increase thermogenesis by enhancing mitochondrial biogenesis and respiration in intramuscular adipocytes of HFD-fed mice 88. Notably, melatonin treatment prevented mitochondrial fission via SIRT1/PGC-1α activation in hyperglycemia-treated cells and streptozocin (STZ)-induced diabetic mice 89. Melatonin is expected to reach maximal plasma levels after 30-60 min in oral administration and 30-45 min in intravenous (IV) administration 90. The elimination half-life (t1/2) for a dose of 0.5-6 mg melatonin in oral administration is approximately 46-65 min, while the t1/2 is about 28-61 min for IV administration of 100 mg melatonin 90, 91. Also, melatonin metabolism acts faster in children than adults. To improve the bioavailability of melatonin, the strategies including subcutaneous injection, oral transmucosal, intranasal, and transdermal can be considered 92.

## Melatonin can possibly halt or even prevent the pathogenesis of T2DM-induced AD-like features

In the previous sections, we have summarized that the mechanisms of metabolic diseases in decreasing cognitive functions may be related to oxidative stress, mitochondrial dysfunction, IR, and inflammation. In this section, we discuss the effects of melatonin in preventing T2DM (or related diseases)-induced AD pathology. As shown in Table 2, different mechanisms of melatonin in treating AD are summarized.

Melatonin can be secreted into the blood, CSF, brain and peripheral tissues. Melatonin (5-methoxy-N-acetyltryptamine) is a pleiotropic hormone derived from vertebrate pineal glands to regulate the circadian and seasonal rhythms, sleep, retinal functions and the immune system 106, 107. The first step of melatonin biosynthesis is the hydroxylation of tryptophan to generate 5-hydroxytryptophan, and then 5-hydroxytryptophan is decarboxylated to produce serotonin. Next, the arylakylamine N-acetyltransferase acetylates serotonin to N-acetylserotonin. Finally, N-acetylserotonin is methylated by O-methyltransferase to generate melatonin 108. The synthesis of melatonin is regulated in a circadian manner. After biosythesis, melatonin is right to be transferred into the cerebrospinal fluid (CSF) and bloodstream. Melatonin exists like a transient state in the body that is rapidly metabolized in the body and its half-life is only around 20-30 min 109. Melatonin has been observed to be a free radical scavenger, immune modulator, and neuroprotectant 110. Melatonin treatment was found to improve cognitive function and reverse sleeplessness in neurodegenerative diseases, through Nrf2 activation and inhibition of proinflammatory cytokines 111. In AD patients, Aβ accumulation and proinflammatory cytokines impair the BBB permeability.

The BBB breakdown, accompanying increased levels of ROS, metalloproteinase (MMP)-2, and IFNγ, could enhance the circulating neurotoxins enter the brain due to selectivity loss, and finally exacerbate AD progression 112. The disruption of BBB has also been observed in T2DM individuals by changing its permeability and integrity 106, 107. In addition, the permeability of BBB increased via alteration of the tight junction protein expression in STZ-induced diabetic rats 113. In obese individuals, the macrophage infiltrated in adipocytes undergo M1 proinflammatory state, leading to excessive secretion of proinflammatory cytokines and chemokines, which can cross the BBB and affect brain functions 114. Melatonin has protective effects in brain microvascular endothelial cell via MMP-9 and nicotinamide adenine dinucleotide phosphate (NADPH) oxidase-2 expression 115, 116.

**Figure 4:**
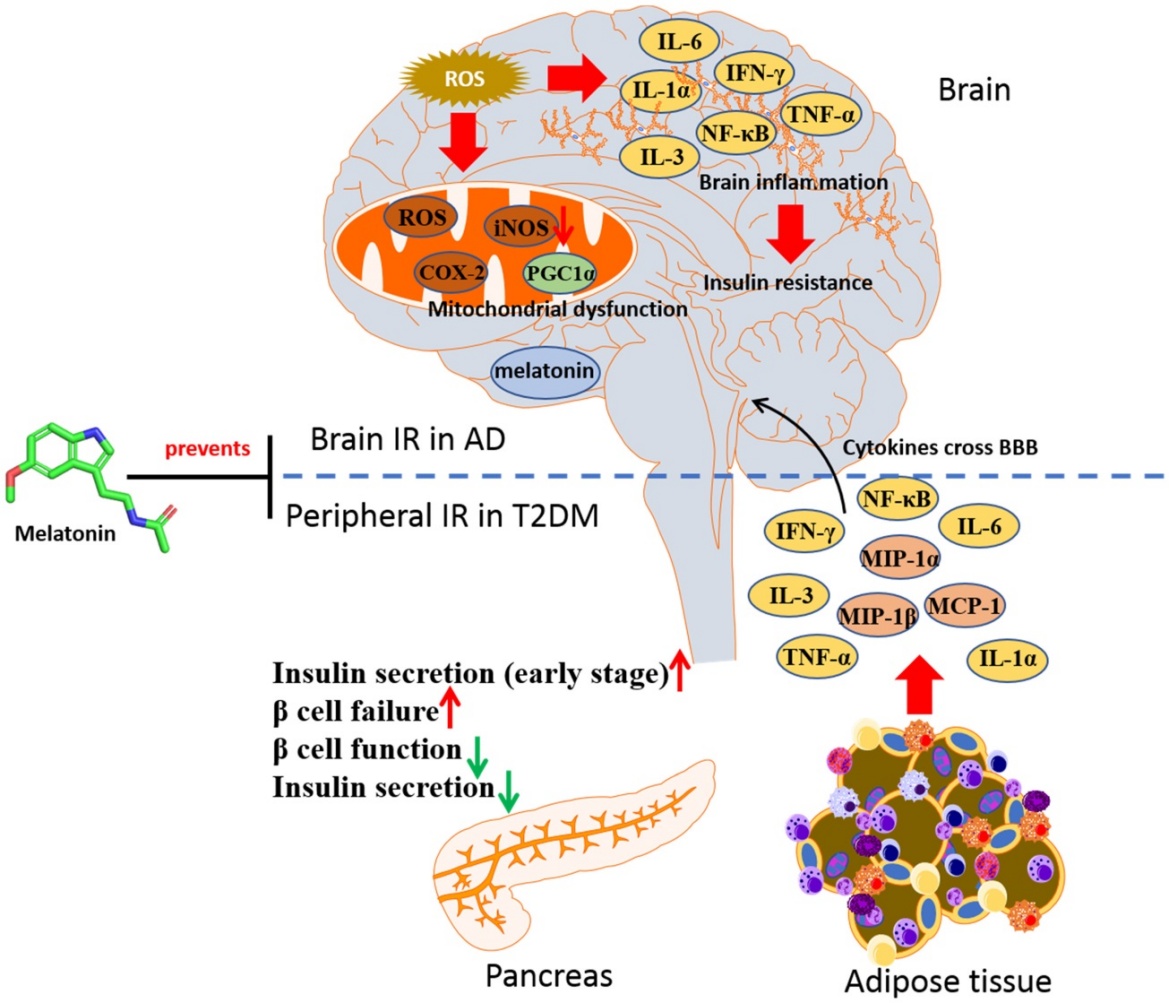
** Melatonin in prevention against insulin resistance of AD and T2DM.** In T2DM, a parallel inflammatory mechanism leads to brain insulin resistance and cognitive dysfunction in AD. Melatonin can be an agent in halt the progression of AD in T2DM by targeting insulin signaling.

In HFD-induced cognitive impairment mice, melatonin was shown to prevent the oxidative stress in hippocampus through decreasing the level of GSSG and increasing GSH/GSSG ratio 117. In STZ-induced rats which displayed AD features, melatonin was effective in decreasing both Aβ formation and tau proteins hyperphosphorylation in hippocampus, as well as reducing the phosphorylation of IRS1 and restoring the phosphorylation of glycogen synthase kinase 3β (GSK3β) 104. These observations indicate that melatonin may be protective in individuals suffering from diabetes and slow down the progression to AD, via restoring insulin signaling. Moreover, the role of melatonin in GSK3β regulation elucidated that GSK3β would interact with presenilin-1 to prevent neurodegeneration in AD 118, 119. Chronic melatonin exposure could attenuate the tau protein hyperphosphorylation via activating PI3K/Akt/GSK3β in Aβ_42_ treated mice. It was also reported that melatonin could prevent T2DM-induced cognitive deficits in rats through anti-neuroinflammatory activity. In the combination of HFD and STZ-induced cognitive dysfunction rats, melatonin treatment was shown to significantly reduce the expression levels of the neuroinflammatory mediated factor including IL-6, TNF-α, iNOS and COX-2, along with inhibiting the expression of NF-κB and IKK phosphorylation, as well as mitigating increasing mitochondrial function 120. Besides, the neuroinflammation found in HFD-induced T2DM rat could be reversed with melatonin treatment, accompanying with repression of iNOS, IDO1, and AChE, indicating that the antioxidant and anti-inflammatory effects of melatonin can be applied in fighting against neuroinflammation associated with T2DM 58. It is belived that melatonin will be a potential strategy in both T2DM and AD therapy through reversing IR (Figure 4).

## Safety of melatonin

Melatonin can act as a broad spectrum antioxidant partially due to its lipophilic and hydrophilic properties, which can cross the barriers easily within subcellular organelles. Although limited data concluded the safety of exogenous melatonin, it can be suggested as a safe drug. Melatonin showed no significant adverse effects on adolescents, children, or preterm infants except high doses or long-time administration 121, 122. In children with epilepsy, a dosage of 9 mg per day for 4 weeks may have adverse effects including headache, diarrhea, hypothermia, dizziness, rash, and gastrointestinal symptoms 123. In seasonally breeding mammals, melatonin was shown to decrease estrogen secretion in long-breeders but increase estrogen levels in short-breeders 124, 125. However, the side effects of melatonin on reproduction of human beings remain unknown. Since the dosage of melatonin in pregnancy subjects is not studied, it is not recommended for pregnant women to use melatonin 126. It is hypothesized that melatonin may affect the ovaries but the exact mechanisms need to be determined 127.

## Conclusion and perspectives

Melatonin acts like a master clock in the suprachiasmatic nuclei (SCN) and is associated with multi-oscillatory network in mammal organisms 128. Based on existing studies, deviant circadian rhythms and poor sleep quality may increase the risk of metabolic and cognitive diseases. Some reports have suggested that endogenous melatonin showed protective effects on endocrine and neurological systems. Melatonin is also important in regulating the secretion of metabolic hormones like leptin and ghrelin, which are key mediators in energy homeostasis. In experimental models of AD, the neurodegenerative symptoms were prevented by melatonin via the removal of toxic proteins by the brain glymphatic system. These studies indicated that melatonin is beneficial in T2DM and AD, although it remains inconclusive whether melatonin treatment in patients might raise any adverse effects.

Numerous studies have demonstrated that T2DM could accelerate and exacerbate AD. There are overlapping mechanisms of T2DM and AD including oxidative stress, mitochondrial dysfunction, IR, and inflammation. IR is a characteristic of T2DM, and a potential indicator of AD. Melatonin seems to meet the criteria of exhibiting highly significant protective actions against these conditions, especially in T2DM induced AD, by targeting the metabolic pathways regulated by brain insulin. Currently, the adverse effects of melatonin is yet to be elucidated in detail but most of the effects of melatonin have been proven to be safe in human and animal models at various dose ranges. Considering the high efficacy of melatonin in increasing mitochondrial bioactivity and insulin sensitivity, it provides an insight to investigate the clinical efficacy and safety of melatonin in halting the progression of AD in T2DM individuals.

## Figures and Tables

**Table 1:** Pharmacological studies of melatonin on different T2DM models.

Pharmacological effects	Mechanisms	Ref.
**Zücker diabetic fatty (ZDF) rats**		
Decreased hyperleptinemia; raised hypoadiponectinemia; improved homeostasis	HOMA-IR↓; insulin resistance↓; serum free fatty acid↓	45
Attenuated low-grade inflammation and oxidative stress	IL-6, TNF-α and CRP↓; inflammation↓; LPO↑; oxidative stress↓	46
Promoted fat browning	PGC-1α and UCP1↑; mitochondrial function↑	47, 48
Reduced the oxidative status; reduced apoptosis susceptibility	Respiratory control ratio (RCR)↑; nitrite levels↓; mitochondrial function↑	35
Prevented impaired functions of hepatic mitochondria in diabetic obese animals	ALT↓; glycogen and lipid accumulation↓; tate 3 respiration and RCR↑; oxidative stress↓; UCP2↓; mitochondrial function↑	49
**Combined of HFD and STZ treated rodents**		
Prevented retinal damage in early T2DM	NOS activity↓; TNFα↓; inflammation↓; oxidative stress↓;	50
Decreased apoptosis; improved cardiac function in T2DM	SIRT1 signaling↑; PERK/eIF2α/ATF4 signaling↓; oxidative stress↓; ER stress↓	51
Restored the vascular responses and endothelial dysfunction in diabetes	Fasting blood glucose, total cholesterol and LDL levels↓; oxidative stress↓	52
Increased lipid peroxidation; reduced hypertension and fatigue syndrome	Lipid profiles↓; serum adiponectin↑; GLUT4, PGC-1α, mTFA and NRF↑; oxidative stress↓; insulin resistance↓	53
**HFD-fed rodents**		
Reduced hyperglycemia	Total cholesterol and triacylglycerols↓; blood glucose↓; insulin resistance↓; oxidative stress↓	54, 55
Decreased hyperleptinemia; raised hypoadiponectinemia;	HOMA-IR↓; insulin resistance↓; glucose tolerance↑	42
Prevented diabetic cardiomyopathy	Caspase-3 and Bax↓; Bcl-2↑; insulin resistance↓; ER stress↓	56
Improved metabolic flexibility	Total cholesterol, triglycerides and LDL-cholesterol↓; IL-6 and TNFα↓; inflammation↓	57, 58
Improved brain glucose homeostasis	GSH↑; oxidative stress↓; AChE, iNOS, IL-6, MCP-1 and TNFα↓; inflammation↓	45
**T2DM patients**		
NA	GPx-1, CAT, GR and SOD-1↑; MDA↓; oxidative stress↓	59, 60
NA	CRP, IL-6 and TNF-α↓; inflammation↓	47
**Pinealectomized rats**		
Increased energy expenditure; increased mitochondrial respiratory	PGC-1α, CREB, AKT and CAMKII↑; mitochondrial biogenesis↑; mitochondrial function↓; insulin resistance↓	61
**Rat insulinoma INS-1 cells**		
Prevented hyperglycemia; rescue β-cell viability	glutathione peroxidase, SOD, glutathione reductase and catalase↑; mitochondrial function↑	62
**PA induced IR primary muscle cells**		
Increased energy expenditure; increase mitochondrial respiratory	UCP3, PGC-1α, CREB, AKT and CAMKII↑ mitochondrial function↑; insulin resistance↓	61

**Table 2:** Pharmacological studies of melatonin on different AD models.

Pharmacological effects	Behavioral or cognitive changes	Mechanisms	Ref.
**Amyloid-beta induced AD rats**			
Improved spatial learning and memory, synaptic plasticity; reduced astrogliosis and synaptotoxicity	Less time to reach the platform in Morris water maze (MWM) test; more efficient in swimming path	GFAP**↓**; Musashi1/Notch1/Hes1**↑**	93, 94
Inhibited neurotoxicity and astrocyte activation	NA	GFAP**↓**; MAP**↑**; Reelin/Dab1**↑**	78
Improved spatial learning and memory	Shorter latency in MWM test; increase of period in the III quadrant, raise of numbers of line crossings in central square arena in open field test; increase in the latency and decreased errors in step‐through test and step‐down test	GSK-3β, caspase-3, Aβ1-42 , BACE1 and p-tau↓; PP2A and Bcl-2↑; mitochondrial function↑	95
Improved memory, hindered anxiety, and attenuated hippocampal cell damage	Increased number of arm entries in Y-maze test; increased number of open arm entries and time spent in open arms in EPM test	SIRT1**↑**; COX2 and TFAM**↑**	80
**Scopolamine induced amnesia mice**			
Improved spatial learning and memory	Shorter escape latency in MWM test; longer latency time in passive avoidance test (PAT)	ChAT, CHT and VAChT↑	96
Recovered cognitive impairment	Shorter escape latency in MWM test; longer latency time in PAT	MBP, BDNF, and TrkB↑	97
Attenuated synaptic dysfunction, memory impairment neuroinflammation	Shorter escape latency in MWM test; increased number of arm entries in Y-maze test	CREB and BDNF ↑; Akt and ERK ↑; GFAP, TNFα and IL6 ↓; JNK, Nrf2 and HO-1 ↓	98
**Tg2576 mice overexpressed APP**			
Ameliorated amygdala-dependent emotional memory	No changes in behavioral tests	PSD95↓; Arc, pCREB and c-Fos↑	99
Activated lymphatic system	NA	Aβ↓	100, 101
**AD transgenic mice**			
Induced cognitive enhancement and brain resilience	Novel object recognition (NOR) test	NF-κB, TNFα, IL-1β↓; amyloid and p-tau↓; Gas6 and SIRT1↑	85
Improved episodic memory; reduced neuroinflammation; inhibited reactive microgliosis	Less time spent exploring the new object	amyloid aggregates↓	102
**STZ-induced AD like rats**			
Prevented memory impairment; downregulated AD-like hyperphosphorylation	Shorter escape latency in MWM test	MDA↓; SOD and GSH‐Px↑; antioxidation function↑	103
Ameliorated memory; prevented brain insulin resistance	Shorter escape latency in MWM test	p-tau, BACE1 and PS1↓; AKT and GSK-3β↑	104
**Aged mice**			
Improved hippocampal neuronal homeostasis	NA	SIRT1, FOXO1, MT1 and MT2↑; p53, ac-p53, MDM2, and DKK1↓	105
